# Pathologic Findings at Risk Reducing Surgery in *BRCA* and Non-*BRCA* Mutation Carriers: A Single-Center Experience

**DOI:** 10.3390/diagnostics12123054

**Published:** 2022-12-06

**Authors:** Chiara Cassani, Chiara Rossi, Cristina Angela Camnasio, Mario Urtis, Giacomo Fiandrino, Maurizia Grasso, Francesca Zanellini, Marco Lucioni, Gioacchino D’Ambrosio, Alessandro Di Toro, Margherita Rossi, Marianna Roccio, Alberta Ferrari, Simona Secondino, Rossella Elena Nappi, Eloisa Arbustini, Marco Paulli, Arsenio Spinillo, Stefania Cesari

**Affiliations:** 1 Department of Clinical, Surgical, Diagnostic and Pediatric Sciences, Unit of Obstetrics and Gynecology, University of Pavia, IRCCS San Matteo Hospital Foundation, 27100 Pavia, Italy; 2 Department of Molecular Medicine, Unit of Anatomic Pathology, University of Pavia, IRCCS San Matteo Hospital Foundation, 27100 Pavia, Italy; 3 Transplant Research Area and Centre for Inherited Cardiovascular Diseases, Department of Medical Sciences and Infectious Diseases, IRCCS San Matteo Hospital Foundation, 27100 Pavia, Italy; 4 Unit of Anatomic Pathology, IRCCS San Matteo Hospital Foundation, 27100 Pavia, Italy; 5 Unit of Obstetrics and Gynecology, IRCCS San Matteo Hospital Foundation, 27100 Pavia, Italy; 6 General Surgery III—Breast Surgery, IRCCS San Matteo Hospital Foundation, 27100 Pavia, Italy; 7 Unit of Medical Oncology, IRCCS San Matteo Hospital Foundation, 27100 Pavia, Italy; 8 Department of Clinical, Surgical, Diagnostic and Pediatric Sciences, Research Center for Reproductive Medicine, Gynecological Endocrinology and Menopause, University of Pavia, IRCCS San Matteo Hospital Foundation, 27100 Pavia, Italy

**Keywords:** serous tubal intraepithelial carcinoma, risk-reducing surgery, *TP53*, *BRCA*, high-grade serous carcinoma, non-*BRCA* mutations, occult cancer

## Abstract

Risk-reducing surgery (RRS) is recommended in BRCA-mutated carriers because of their increased risk of developing ovarian cancer, while its role is still discussed for women harboring mutations in non-*BRCA* homologous repair genes. The aim of this study was to retrospectively evaluate the occurrence of pathological findings in a high-risk population undergoing RRS in San Matteo Hospital, Pavia between 2012 and 2022, and correlate their genetic and clinical outcomes, comparing them with a control group. The final cohort of 190 patients included 85 *BRCA1*, 63 *BRCA2*, 11 *CHEK2*, 7 *PALB2*, 4 *ATM*, 1 *ERCC5*, 1 *RAD51C*, 1 *CDH1*, 1 *MEN1, 1 MLH1* gene mutation carriers and 15 patients with no known mutation but with strong familial risk. Occult invasive serous carcinoma (HGSC) and serous tubal intraepithelial carcinoma (STIC) were diagnosed in 12 (6.3%) women, all of them *BRCA* carriers. No neoplastic lesion was diagnosed in the non-*BRCA* group, in women with familial risk, or in the control group. Oral contraceptive use and age ≤45 at surgery were both found to be favorable factors. While p53 signature and serous tubal intraepithelial lesion (STIL) were also seen in the control group and in non-*BRCA* carriers, STIC and HGSC were only found in *BRCA1/2* mutation carriers.

## 1. Introduction

High-grade serous carcinoma (HGSC) is the most common type of epithelial ovarian cancer (EOC). It is an aggressive malignancy, carries a poor prognosis and represents over 70% of all EOC-related deaths. As most HGSC cases present as advanced disease, it is often unclear where HGSC truly originates. Traditionally, the ovary surface epithelium was believed to be the site of origin, but epidemiological and molecular evidence has shown that a subset of HGSCs do not arise from the ovarian epithelium, but rather from the fallopian tube epithelium [1,2,3,4]. Particularly, serous tubal intraepithelial carcinoma (STIC), a noninvasive neoplastic lesion developing preferentially from the secretory cells of the distal fallopian tube epithelium, has been proposed as a precursor for a subset of HGSC [5]. From a histological point of view, STIC is defined by the combination of an atypical cell morphology, an aberrant immunohistochemical expression of p53 (either strong diffuse expression or absent/“null” staining, or other aberrant patterns) and an increased proliferation rate (as measured by Ki-67/MIB1 immunohistochemistry). Recent data suggests that STIC can progress to invasive carcinoma and even metastasize through a process defined in literature as “precursor escape” [6].

STIC is not the only p53-aberrant lesion developing in the tubal epithelium, and rather represents only the final, overtly preneoplastic end of a spectrum of alterations that have been described in the tubal fimbric epithelium. A P53 signature is defined as a morphologically normal continuous length of non-ciliated (secretory) cells with an intense, aberrant immunohistochemical staining for p53, compatible with an underlying mutation of the gene, and a low proliferative index [7]. A spectrum of histological changes fills the gap between the clinically harmless p53 signature and STIC; among these, serous tubal intraepithelial lesion (STIL) [6] or “serous tubal epithelial lesion/proliferation of uncertain significance” represents a more heterogeneous ensemble, usually falling short of the degree of cytological and architectural atypia needed to define STIC, but characterized by a p53 staining pattern compatible with an underlying mutation. On the opposite end of the spectrum, secretory cell outgrowth (SCOUT) represents a probable precursor lesion of p53 signature, usually located at the proximal end of the salpinx, defined as nests of at least 30 secretory epithelial cells with pseudostratification, low mitotic activity and no immunohistochemical aberrant expression of p53 or alterations of *TP53* gene [8].

Despite sharing *TP53* mutations with HGSC, there is currently no definitive evidence of progression of these lesions, moving from an initial hit with p53 signature, with progression through STIL and STIC, with HGSC as the terminal event. However, STIC has been found to harbor the same *TP53* mutations of the concurrent HGSC, and the presence of shared mutations in the p53 signature may hint to the existence of this carcinogenesis [9].

The current theory is that the p53 signature-STIC axis may represent one of the pathways of onset of HGSC, but that an additional contribution from the tubal microenvironment is still needed to gain the molecular alterations driving the carcinogenesis.

The hypothesis of a pathway other than STIC-to-HGSC still remains [4], especially to explain those cases in which no tubal precursor is identified despite extensive tubal histological sampling; molecular studies have hinted that HGSC may in some cases arise from ovarian low-grade serous carcinoma and ectopic fallopian tube epithelium located in the ovary, such as endosalpingiosis and cortical inclusion cysts [10,11,12,13].

The identification of the fallopian tube preneoplastic lesions, and the insight on the biological events leading to the onset of HGSC has largely been possible through the histopathological analysis of adnexectomies performed on healthy patients with a genetic susceptibility or a strong familial history of ovarian (and breast) cancer as a primary prevention for the development of HGSC.

According to the current knowledge [14,15], 20–30% of HGSC have a genetic etiology; in these women, risk-reducing surgery (RRS) represents the only primary prevention for ovarian cancer. The risk of developing the disease varies according to the mutated gene and the penetrance of the specific mutation [16,17,18].

Mutations that confer a higher risk of developing ovarian cancer usually come from the Homologous Recombination Repair (HHR) pathway associated with Hereditary Breast and Ovarian Cancer Syndrome or mutations in mismatch Repair genes associated with Lynch Syndrome. Pathogenic variants in *BRCA1* and *BRCA2* genes are the most frequent mutations in the general population and are known to be related with a lifetime risk of developing EOC of 44% and 17%, respectively [19]. In these women, RRS for EOC prevention reduces mortality and is recommended to date by all international scientific societies [20].

In recent years, the expanding use of multigene panels for the assessment of cancer susceptibility has allowed the identification of mutations of moderate or possibly low penetrance for which no established management guidelines exist yet to optimize patient care.

In fact, while the real magnitude of EOC risk related to moderate and low penetrance mutations is not yet well established, the impossibility of an effective ovarian cancer screening warrants consideration of RRS. Currently, the National Comprehensive Cancer Network guidelines (NCCN^®^) [21] state to consider RRS for patients affected by mutations in *BRIP1*, *RAD51C* and *RAD51D*, after childbearing or between 45 and 50 years of age. There is another subset of genes within the HHR pathway or in the Fanconi anemia core complex or involving checkpoint control, such as *PALB2*, *BARD1*, *CHEK2* for which surgery is not yet recommended by guidelines, although the recommendations state to always take into consideration family history when deciding whether RRS is indicated.

Moreover, also for high-risk patients (i.e., patients with clinical criteria for a potential hereditary cancer syndrome, first or second degree relative with ovarian cancer, patients with high-risk breast cancer (BC) criteria) without an identified genetic mutation, a thorough risk stratification can be performed to guide screening and preventive measures, even if surgery remains the only effective strategy [22].

A thorough RRS should consist of a first surgical step, with removal of both adnexa, eventual hysterectomy or at least endometrial biopsy (even if not yet formally recommended by guidelines), and laparoscopic exploration of the peritoneal cavity with peritoneal washing. The second, just as pivotal step is the pathological evaluation of the collected samples, preferentially carried out by an expert pathologist applying the Sectioning and Extensively Examining the FIMbriated End (SEE-FIM) protocol [23].

The aim of this study is to describe the incidence of preneoplastic lesions and occult carcinoma in a high risk population (*BRCA* carriers, carriers of non-*BRCA* gene variants and high risk patients based on family history) who underwent RRS and in a control group.

Moreover, we aimed to investigate the effects of protective and risk factors on development of preneoplastic and neoplastic lesions and treatment and prognosis of occult cancer diagnosed at RRS.

## 2. Materials and Methods

We performed a retrospective analysis of prospective recorded data of all high risk patients who underwent RRS at the IRCCS Policlinico San Matteo Foundation of Pavia between February 2012 and October 2022. Criteria for defining a high risk patient were based on the “Seven-Question family history screening” [24,25] and included one or more of the following: (1) known pathogenic germline variant in *BRCA1* or *BRCA2* genes, (2) known pathogenic germline variant in moderate or low penetrance EOC susceptibility genes, (3) first or second degree relative with EOC and (4) personal history of BC and any degree relative with EOC on the same side of the family. All high-risk patients underwent multi-gene panel testing for inherited cancer syndrome (TruSight Cancer Sequencing Panel–Illumina) based on Next Generation Sequencing (NGS) technology targeting 94 genes and 284 single nucleotide polymorphisms. All pathogenic and likely pathogenic variants were validated using Sanger sequencing according to clinical practice and international guidelines.

All women attended the high-risk clinic and underwent full clinical assessment and trans-vaginal ultrasound to exclude EOC. Preoperative serum CA125 values were obtained for most women. Patients were excluded if there was a preoperative suspicion of cancer. All surgical procedures were performed by an expert surgeon using a laparoscopic approach, both standard or single-site technique according to surgeon choice. In all cases, careful inspection of all peritoneal surfaces was performed. Peritoneal washings for cytology using warm physiologic saline were obtained before any major surgical manipulation. All patients underwent bilateral salpingectomy and/or bilateral oophorectomy and/or total hysterectomy and/or endometrial biopsy according to their surgical consent. The specimens were retrieved using surgical bags.

The control group was represented by an age-matched group of patients who underwent bilateral salpingectomy and/or bilateral salpingo-oophorectomy (BSO) and/or total hysterectomy for benign gynecological pathologies, without personal and family history of BC and EOC.

The BSO specimens were handled according to the SEE-FIM protocol [23]: the fimbriated end was amputated and sagittally sectioned at 2-mm intervals, and the remaining portion of the tube was cross-sectioned longitudinally; the ovary was entirely submitted. Hysterectomy specimens were extensively sampled; the entire endometrial cavity and at least two sections of cervical canal were embedded and examined [26]. Endometrial biopsies were entirely submitted and examined at least at two levels of section. Histological slides were evaluated by one or more dedicated gynecopathologists (SC, GF, GDA).

Immunohistochemical staining for p53 (clone DO7, monoclonal, Dako) and Ki67 (clone MIB1, monoclonal, Dako) were performed on fimbriae sections of both cases and controls. Elevated MIB1 index (>10% nuclear staining) and abnormal p53 staining (overexpression, complete loss of expression or other abnormal patterns) were used as supportive evidence for the diagnosis [27].

P53 signature was defined as ≥12 histologically inconspicuous cells with abnormal p53 pattern and a low MIB1 labeling index. STIL was defined as a lesion characterized by abnormal p53 staining, variable MIB1 labeling and initial architectural atypia, with lack of ciliated cells, increased nuclear-to-cytoplasmic ratio, usually with preserved polarity of the epithelium and without striking cellular atypia; it also included those lesions that fell short of the diagnosis of STIC. STIC was chiefly diagnosed on epithelial morphologic evaluation alone: increased nuclear/cytoplasmic ratio, nuclear pleomorphism, epithelial stratification with loss of polarity, irregular epithelial thickness and exfoliation of cells into tubal lumen, with immunohistochemistry as an ancillary, supporting evidence (aberrant p53 pattern of staining, high MIB1 labeling index).

**Figure 1:**
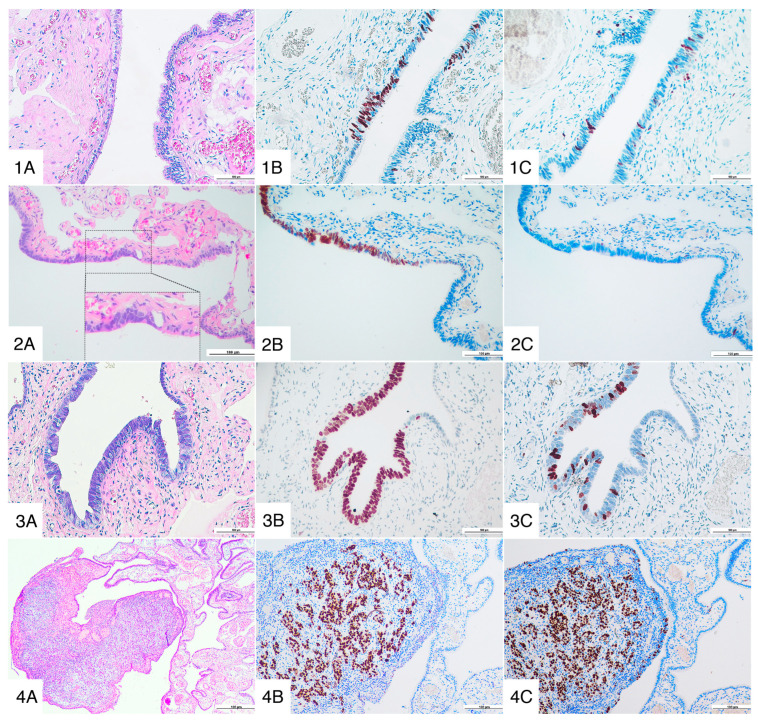
(1) p53 signature histology (**1A**, HE, 10×), p53 immunohistochemical staining (**1B**, 20×) and Ki-67/MIB1 index (**1C**, 20×); (2) STIL histology (**2A**, HE, 20×) with the inset showing the transition between the normal ciliated tubal epithelium on the right-hand of the picture, and the lesion on the left-hand of the inset, p53 immunohistochemical staining (**2B**, 20×) and Ki-67/MIb1 index (**2C**, 20×); (3) STIC histology (**3A**, HE, 20×), p53 immunohistochemical staining (**3B**, 20×) and Ki-67/MIB1 index (**3C**, 20×); (4) invasive high grade serous carcinoma of the fallopian tube (**4A**, HE, 4×), p53 immunohistochemical staining (**4B**, 10×) and Ki-67/MIB1 index (**4C**, 10×).

Occult cancer was defined as clinically inapparent invasive malignancy of the epithelium of the ovary or fallopian tube diagnosed at histopathological examination, according to the guidelines of the International Federation of Gynecology and Obstetrics (FIGO 2018) [28] (Figure 1).

Data regarding genetic variants, oncologic personal and family history, age at genetic test and surgery, prior chemotherapy, oral contraceptive (OC) and tamoxifen use, menopausal status at surgery and parity were collected. Type of RRS, histology of the surgical specimen, surgical outcomes, cancer status and last follow up were also recorded.

Statistical analysis was performed using the SPSS software version 25 and *p* values < 0.05 were deemed to be significant. Absolute and percentage frequencies were used to describe categorical patients’ population while mean values and ranges were used for continuous variables. Fisher’s Exact test was used for testing association between categorical variables and two-tailed t-test was used for continuous variables.

## 3. Results

From 2012 to 2022, 190 consecutive high risk women were referred for RRS to the Gynecology Department at Fondazione IRCCS Polyclinic San Matteo, Pavia. Of these, 148 were *BRCA1* or *BRCA2* mutation carriers, 27 were carriers of pathogenic variants of non-*BRCA* genes, and 15 had no pathological gene mutations but fulfilled the criteria for patients at high risk for EOC.

Across the same time period, we also enrolled 145 consecutive patients who underwent BSO or salpingectomy for non-risk reducing purposes and had a silent oncological history, representing our control group.

Characteristics of women and surgery are summarized in Table 1 and Table 2.

In the high risk population, mean age at genetic testing was 48 years (range 26–79) and 124 out of 190 (65.3%) were postmenopausal. The majority of them had a personal history of BC (*n* = 116; 61.1%) and nearly one third (*n* = 52; 27.4%) had used tamoxifen. The use of OC was reported by 81 patients in the high-risk group (42.6%).

At genetic testing, pathogenic or likely pathogenic variants were identified in 91.6% of patients: 85 were *BRCA1*, 63 were *BRCA2*, 11 were *CHEK2*, 7 were *PALB2*, 4 were *ATM*, one *ERCC5*, one *RAD51C*, one *CDH1* and one *MEN1*.

Mean age at RRS was 49 and concomitant hysterectomy was performed in 33.15% (*n* = 63) of cases, while 25.26% of patients (*n* = 48) underwent endometrial biopsy at the time of surgery and in most cases peritoneal cytology was also performed.

Only one intraoperative complication (0.5%) and three (1.6%) transient post-operative complications were reported, as seen in Table 2. In no case was conversion to open surgery required.

The control group consisted of 145 BSO whose mean age at surgery was comparable (50 years of age) to the high-risk group. The vast majority of patients in the control group (70.3%) underwent concomitant hysterectomy because of benign uterine disease (genital prolapse, uterine myomas, cervical dysplasia, ovarian cysts, endometriosis) or gender dysphoria. Three intraoperative (2.1%) and one postoperative (0.7%) complications were reported as seen in Table 2, all resolved without sequelae.

Table 3 details histological findings of our cohort.

Briefly, of 190 high risk patients undergoing RRS, 12 (6.3%) were diagnosed with neoplastic lesions (7 invasive serous carcinomas (3.7%), 8 STICs (4.2%), of which 3 had both) while no occult tubo-ovarian neoplasia, both invasive or in situ, was identified in the control group (*p* = 0.0011). All STICs and occult cancers were found in *BRCA1* and *BRCA2* carriers and clinical and histological information are summarized in Table 4.

Notably, no STICs or invasive malignancies were found in patients undergoing RRS for familial risk or non-*BRCA* gene mutations. Of note, a patient with a *CHEK2* mutation was diagnosed with a stage IA serous borderline ovarian tumor, a lesion that has been suggested to belong to an ovarian carcinogenesis pathway alternative to the STIC-to-HGSC progression, as previously mentioned.

The presence of preneoplastic and neoplastic lesions in *BRCA* mutated patients was significantly lower in patients with a history of OC use than in never users (1.5% vs. 13.4%; *p* = 0.012).

While postmenopausal status at surgery was not significantly associated with the diagnosis of STIC or HGSC (*p* = 0.74), physiological menopause highly increased the frequency of malignant and pre-malignant lesions with respect to both chemo- and pharmacological-induced menopause or being premenopausal (18.6% vs. 5.4% vs. 0% vs. 5.8%; *p* = 0.04864). Parity, tamoxifen use, personal history of BC, previous chemotherapy and familial history of EOC or BC were not significantly associated with a diagnosis of preneoplastic and neoplastic lesions.

Of the 7 patients with occult carcinoma, 4 patients had a documented tubal origin with identification of STIC in the fimbrial portion in 3 cases, whereas in the remaining 3 cases the fallopian tube was spared.

Peritoneal washing was performed in all cases and neoplastic cells were detected in 4 out of 12 patients (33.3%), mostly in invasive cancers. Only one *BRCA1* mutated patient was found with STIC and positive peritoneal washing at the time of RRS including BSO and total hysterectomy. She underwent thoracic and abdominal CT scan without evidence of disease, and she underwent a second look surgery with peritoneal washing and biopsies 6 months after the diagnosis, all negative. She was strictly followed by CA125 testing, pelvic ultrasound and annual CT scan and she is currently disease-free 4.5 years after diagnosis, as are all other patients with STIC and negative cytology (mean follow up period of 68.6 months; range 35–155 months).

All patients with invasive neoplasm were staged, debulked and treated according to current guidelines: 50% of patients were at advanced stages (stage III) at diagnosis and 2 patients experienced relapse (one stage IIIC and one stage II) and are currently in treatment with Poly ADP Ribose Polymerase inhibitors (PARPi).

Of note, mean CA125 at surgery in invasive/STIC cases was 25.4 U/mL, significantly higher compared to 7.3 U/mL of *BRCA* patients without neoplastic lesions (*p* < 0.00001).

One ovarian cancer patient was also diagnosed with concomitant endometrial endometrioid carcinoma. Another endometrioid endometrial invasive cancer and two intraepithelial endometrial neoplasms (EIN) were diagnosed in *BRCA* mutated women who opted for hysterectomy at the time of RRS, in the absence of other indication and without any other pathological findings at surgery. The frequency of p53 signature and STIL was similar in both high-risk population and controls (10.2% and 8% vs. 9.7% and 12.4%, respectively), without significant difference among *BRCA* and non *BRCA* gene mutation carriers and patients with familial risk.

## 4. Discussion

The most important issue to be raised when discussing the pathological findings at RRS is the wide range of results existing in the literature regarding the incidence of the different types of lesions (p53 signature, STIL, STIC). As we will detail further, no real concordance exists about the actual incidence of any of these lesions, and even the rate of occult carcinoma and STIC varies significantly depending on the considered study. This divide may be partly ascribed to the heterogeneity of background, that comprises both single-center experiences, as the present work, and larger reviews and metanalyses, but also to the lack of reproducibility in diagnosing precursor lesions.

The process starts with gross examination: all the lesions that we have discussed are grossly unapparent. The SEE-FIM protocol has been demonstrated to increase the diagnostic yield, by maximizing the fimbrial surface visible at histology [23,29]; however, even in the presence of a thorough grossing, clinical indications and the pathologist’s experience in the field play an important role in recognizing these lesions.

Microscopic evaluation is particularly tricky for small inapparent lesions like p53 signature and STIL, which are easily missed if the immunohistochemical staining for p53 is not performed or is qualitatively unsatisfactory. Even though p53 immunohistochemistry as a “surrogate” of *TP53* status has been demonstrated to be specific, sensitivity varies depending on the underlying mutation, with the “null” pattern of staining (associated with truncating mutations) representing a possible cause of underestimation in up to a third of cases [9,30,31]. The need for additional markers to improve diagnosis sensitivity is under investigation (p16, PAX2, stathmin, laminin γ1, cyclin E1, among others) [32,33]. Concerning STIL, it must be noted that the degree of cytoarchitectural atypia needed for the diagnosis, falling short of a diagnosis of STIC, is vague; no standardized definition exists for a diagnosis of certainty, thus limiting reproducibility. Algorithms have been proposed to guide the pathologist in the diagnosis of the *TP53*-related lesions [27], that aim to reach a satisfactory degree of reproducibility in the diagnosis; however, their widespread application is still lacking, and the concordance in this field, even for STIC, is low, with reported Cohen’s *κ* values between 0.33 and 0.68 [6,27,34,35].

Thus, specific training, close integration between clinical history and pathological findings, and more widespread standardization of the diagnostic algorithm are needed to reduce heterogeneity in this field. This will help the clinicians to make better informed-decisions about the management of precursor lesions in women who are, in most cases, healthy and undergoing risk-reducing (not curative) surgery.

We found an incidence of 3.7% (*n* = 7/190) of occult invasive ovarian and tubal carcinoma among our population; all the invasive lesions arose in carriers of *BRCA1/2* genes mutations, whereas none of the moderate- and low-penetrance non-*BRCA* gene mutation carriers, familial risk patients, and women in the control group showed invasive lesions. Of these invasive carcinomas, 3 (42.8%) had an ovarian origin without any precursor lesion identified in the fallopian tube. This can be explained, as already mentioned, by the fact that STIC can be easily missed at the grossing stage and, even when the SEE-FIM protocol is correctly applied, only a small fraction of the tissue is actually sectioned and examined microscopically, and smaller lesions can be easily left uncut inside the embedded block; moreover, the alternative pathway of origin, from the ovarian epithelium or endosalpingiosis foci in the ovary and peri-adnexal tissues, should also be taken into account.

Our study spans a 10-year period and highlights a risk of occult invasive carcinoma in the lower range of what is reported in literature for high-risk women undergoing RRS (2–14%) [36,37,38,39,40,41]; however, this risk is still higher than in the control group. The “age factor” is probably one of the main contributors for the higher incidence of occult carcinoma with respect to the general population: the median age of our high-risk population was 49 (range 27–79), much higher than the age recommended by international guidelines for RRS (before 40 for *BRCA1*, and before 45 for *BRCA2*) [21,42]. In our study, 65 out of 81 (80.2%) *BRCA1* carriers and 37 out of 63 *BRCA2* carriers (58.7%) underwent surgery beyond guideline recommended ages and the detection of pathologic findings on histology was significantly higher in those older than 45 (age > 45 years = 35/114 vs. age ≤ 45 years = 7/66; *p* = 0.0019).

The apparent delay in RRS in our cohort is partially explained by more advanced age at genetic testing despite a positive personal and familial history for BC and EOC.

Regarding management of patients incidentally detected with occult EOC at the time of RRS, they were treated according to stage with surgery and medical therapies, according to current guidelines. Patients’ outcomes were those expected based on disease stage and *BRCA* mutation status, as highlighted in a recent study by Cowan et al. [43]. The latter found that there is no favorable prognosis of occult disease compared to incidental disease as suggested by other authors [44]. Thus, our data both support the clinical indication for RRS in high-risk patients and underline the importance of closely adhering to the guideline-recommended age groups.

In our results, no occult neoplasm was diagnosed in non-*BRCA* patients. The only relevant diagnosis was the serous borderline ovarian tumor in the *CHEK2* mutated patient, who underwent RRS for her genetic mutation as there was no preoperative suspicion of cancer, no personal history of BC or family history of EOC.

In a recent prospective observational study, the largest single institution series including 27 non *BRCA* gene mutation carriers, Rush et al. [40] reported a diagnosis of STIC in a carrier of a *PALB2* mutation with a strong family history of both BC and EOC; another case report by Gregory-Davis et al. [45] reported a diagnosis of STIC in a *RAD51D* mutation carrier; HGSC and a low grade serous carcinoma were reported by Schoolmeester et al. [46] in two patients, carrying mutations in *RAD51C* and *CHEK 2,* respectively. All these results must be taken with caution, because these mutations have a very low incidence, and large case-control studies are needed to validate the findings; however, we still cannot exclude the possibility of these genes conferring a moderate risk of developing EOC [47]. Their increasing presence in multigene panels in clinical practice will surely help shed light on the actual risk that these mutations carry. Waiting for more solid data, we encourage RRS for moderate penetrance gene carriers in light of the lack of effective screening tools for EOC and of low rate of surgical complications.

The incidence of STIC reported in the literature varies between 0.2% and 11.5% [3,30,48,49,50], and it is almost invariably associated with *BRCA1/2* somatic or germline mutations. Our results are in line with these findings, with a frequency of 4.2% (*n* = 8/190) in the high-risk cohort and 0% in the control group, with all cases reported in women with *BRCA1/2* mutations and none in the moderate-to-low penetrance non-*BRCA* genes or familial risk women.

As of today, there are no specific guidelines for surgical staging or pharmacological treatment of patients with an incidental diagnosis of STIC. All our patients underwent close follow up, without restaging procedures or chemotherapy, according to suggestions by the ESMO-ESGO consensus conference [51] and to published evidence [38]. However, because of the strong association between STIC and *BRCA* mutations and in light of the extremely low frequency of incidental STIC diagnosis in a low-risk population [52], a diagnosis of STIC in patients with silent or missing personal and family history warrants a genetic consultation with evaluation of *BRCA* mutational status.

The risk of developing peritoneal serous carcinoma after RRS is reported to be as high as 4.3% in the literature [53,54]. In our series of patients, no STIC diagnosis has been followed by the development of peritoneal implants or carcinomatosis. In this instance, the literature strongly emphasizes the importance of adhering to strict and standardized follow-up protocols.

P53 signature is considered a precursor lesion in the carcinogenesis of HGSC. Our data show a p53 signature frequency of 10.2% (*n* = 19/190) in the case study population and of 9.7% (*n*= 14/145) in the control population. Our data are in line with the literature (18% in *BRCA* carriers and 15% in controls by Sina et al. [55], 10% in low risk population by Nishida et al. [56], 20% in *BRCA* carriers and 25% in controls by Shaw et al. [57]).

To the best of our knowledge, no study addresses the issue of incidence of p53 signature and STIL in low- and moderate-penetrance non-*BRCA* gene mutation carriers or in patients with familial risk of EOC only.

The frequency of p53-related lesions in the healthy controls and in the high-risk population without *BRCA1/2* gene mutation was comparable to that of *BRCA* carriers, but to date, the data show no progression towards HGSC; no STIC or invasive carcinoma was reported in either group. Our observations support the hypothesis that p53 signature may be a common event in every woman′s life, is independent from the presence of germline mutation in susceptibility genes and therefore may not represent a significant precursor lesion in the vast majority of cases. The hypothesized progression from p53 signature to HGSC is a long one: current evidence supports the assumption that progression from p53 signature to STIC may require up to 20 years, with 6–7 additional years needed for the progression from STIC to HGSC [8,55]. Therefore, our results are aligned with the current literature in suggesting that p53 signature may be a recurrent event in HGSC carcinogenesis, but not sufficient *per se* to trigger a neoplastic transformation. Most likely, other hits, either genetic, environmental or physiological, are required to modulate its oncogenic potential; recent evidence suggests that ovulation, with the monthly release of the reactive oxygen species-rich liquid of ruptured follicles, may be partly responsible for the transformation of the secretory cells at the fimbriated end, by inducing double-strand breaks [4,58,59]. Germline mutations in genes of DNA repair, such as *BRCA1/2*, may just be the substrate necessary to lead the progression of a stochastic mutation to an invasive HGSC. This could explain, at least partly, why those factors reducing the number of ovulatory cycles (i.e., prolonged OC use, multiple parity, duration of breastfeeding) have been demonstrated to have a protective role against HGSC. Similar conclusions can be inferred from our data regarding STIL incidence in the case study population and in controls. No significant difference was found between the two groups (8.0% vs. 12.4%); a breakdown of the case study population reveals a 7.1% incidence in *BRCA1* women, a 9.5% incidence in the *BRCA2* women, no cases in non-*BRCA* gene mutations group and a 20% incidence in women with familial history. In this last group and in the controls, despite the higher or comparable frequency of diagnosis, no STICs or occult invasive carcinoma was diagnosed. This may suggest that STIL, despite displaying an added cytoarchitectural atypia with respect to p53 signature, may represent a lesion that still lacks the molecular potential to progress to a more aggressive disease, and may benefit from the same protective factors.

In this field, the protective role of OC use is well known and supported by studies and meta-analyses both in general [60] and in BRCA-mutated population [61]. In January 2022, a case control study was published [62] with an in-depth analysis of 1733 matched pairs of *BRCA1* or *BRCA2* mutated women that confirmed the protective impact of OC. Cases were less likely to have a history of OC use (with a *p*-value < 0.0001) and even implant use (*p* = 0.001). Any type of hormonal contraception caused a 38% lower risk of developing ovarian cancer in adjusted and non-adjusted models. Our analysis also showed the significant protective benefits of OC (*p* = 0.014) against neoplastic lesions, both invasive or in situ. This makes it of utmost importance to keep discussing OC as an effective option with high-risk patients requiring contraception.

Similarly, physiological menopause is featured by higher rate of ovulatory cycles compared to iatrogenic menopause, both induced by chemotherapy and endocrine therapy. In fact, the literature [58,63] supports the proposal that the lifetime number of ovulatory cycles is directly linked with the risk of developing HGSC and our data supports this evidence. Several hypotheses can explain the link between monthly ovulation and EOC: genomic instability caused by repetitive wounding and healing of the ovarian surface, gonadotropins stimulation of epithelium, repeated exposure to oxidative damage by retrograde menstruation and inflammatory factors of follicular fluid [64], just to cite the main ones.

In depth understanding of multifactorial cancerogenesis in EOC might highlight links between the incessant ovulation and the tubal hypothesis, with important implications in the preventive setting.

A last note must be made regarding endometrial evaluation in this cohort; in our population, 4 (2.2%) occult endometrial lesions (EIN and invasive endometrial carcinoma) were diagnosed, and 4 (2.8%) were diagnosed in the control population. Conflicting data exist about the risk of endometrial cancerogenesis in the *BRCA* population and its magnitude, with some studies suggesting an increased incidence of aggressive endometrial cancer and others negating this [65,66].

Currently, no guidelines address the opportunity of endometrial staging in high-risk patients at the time of RRS; we suggest that an endometrial biopsy or hysterectomy, according to patient age and preference, should be routinely considered in the work-up of these patients, because it increases the chances to identify occult or in situ endometrial neoplasms before they become clinically manifest. Endometrial biopsy at RRS also represents a baseline for subsequent endometrial evaluations.

The main strengths of our study reside in the large number of consecutive high-risk patients enrolled, the availability of a long follow-up and evaluation of CA125 at baseline in almost all of the patients; from the pathological point of view, we stress the presence in our hospital of dedicated gyneco-pathologists handling the grossing and reporting of RRS specimens and the availability of endometrial and peritoneal washing results.

The main limitation of our study is the presence of non-genetically tested patients in the control group, even if we selected patients without personal or familial oncologic history with a very low supposed risk of being carriers of susceptibility gene variants. Another limitation is the relatively small sample size of the non-*BRCA* gene carrier population; this is, however, comparable with other published series and justified by the lack of specific guidelines for the management of these genetic variants.

## 5. Conclusions

In our study of RRS, never OC use, later-than-recommended age at RRS and physiological menopause correlate positively with neoplastic lesions in the *BRCA* population.

At histology, STIC and occult tubo-ovarian carcinoma diagnoses are more frequently associated with *BRCA1/2* mutations, while no definitive conclusion can be drawn regarding the real risk for women with low-to-moderate penetrance non-*BRCA* gene variants. The ever-growing implementation of NGS techniques in clinical practice, and the lack of a viable alternative to surgery, make the need for unequivocal guidelines for non-*BRCA* mutation carriers all the more urgent. Our results underline the fact that data about *TP53*-associated lesions in this population is missing, and we advocate the importance of large case-control studies to reach the statistical significance needed to guide the clinical decision-making process for these patients.

## Figures and Tables

**Table 1:** Baseline and clinical features of study population according to mutation status.

	*BRCA1*	*BRCA2*	Other Genes	Family Risk	Total	Controls
N (% on overall study population)	85	63	27	15	190	145
Mean age at genetic test (range)	46.9 (26–79)	48.01 (31–71)	51.29 (39–77)	47.38 (39–53)	47.94 (26–79)	–
BMI mean (range)	23.93 (17–37)	23.80 (17–38.3)	22.21 (18–32)	23.55 (18–33.6)	23.61 (17–38.3)	25.06 (15–40)
OC use (%)	33 (39.28)	30 (47.6)	9 (33.3)	6 (40)	81 (42.63)	48 (31.1)
History of BC (%)	49 (58.33)	47 (74.6)	10 (37.03)	10 (66.66)	116 (61.05)	0
Tamoxifen use(%)	16 (19.04)	27 (42.85)	4 (14.81)	5 (33.33)	52 (27.36)	0
Previous chemotherapy (%)	38 (45.23)	36 (57.14)	7 (25.92)	5 (33)	86 (45.26)	0
Parity						
0	21 (24.7)	17 (26.98)	6 (22.22)	7 (46.66)	51 (26.84)	34 (23.44)
1	19 (22.3)	17 (26.98)	9 (33.33)	1 (6.66)	46 (24.21)	43 (29.65)
≥2	45 (52.9)	29 (46.03)	12 (44.44)	7 (46.66)	93 (48.95)	68 (46.89)
Menopausal status at surgery						
Premenopause (%)	35 (41.11)	16 (25.39)	10 (37.03)	5 (33.33)	66 (34.73)	92 (63.44)
Postmenopause (%)	50 (58.82)	47 (74.60)	17 (62.96)	10 (66.66)	124 (65.26)	53 (36.55)
Relatives with OC (%)	46 (54.11)	27(42.85)	20 (74.07)	10 (66.66)	103 (54.21)	0
Relatives with BC (%)	65 (76.47)	53 (84.12)	15 (55.55)	8 (53.33)	141 (74.21)	0
Basal CA 125 U/mL mean (range)	10.47 (2–149)	8.86 (2–74)	7.02 (3.5–13.6)	8.27 (3.1– 21.8)	9.27 (2–149)	–

**Table 2:** Surgical characteristics of the study population.

	*BRCA1* (*n* = 85)	*BRCA2* (*n* = 63)	Other Genes (*n* = 27)	Family Risk (*n* = 15)	Total (*n* = 190)	Control (*n* = 145)
Mean age at surgery (range)	48.35 (27–79)	49.31 (35–72)	52.44 (39–77)	50.2 (42–57)	49.4	49.74
Duration of surgery (min), median (range)	91.31 (40–190)	76.70 (25–160)	81.29 (30–165)	78.86 (45–120)	84.02 (35–190)	117 (35–300)
EBL ml mean (range)	65.62 (0–300)	68.73 (0–300)	66.29 (50–200)	56.66 (0–100)	66.06 (0–300)	181 (0–1200)
Concomitant hysterectomy	33 (38.82)	23 (36.50)	6 (22.2)	1 (6.66)	63 (33.15)	102 (70.34)
Endometrial biopsy	14 (16.47)	14 (22.22)	15 (55.56)	5 (33.33)	48 (25.26)	2 (1.37)
Peritoneal washing	75 (89.28)	58 (92.06)	26 (96.29)	13 (86.66)	172 (90.53)	18 (12.41)
Intraop complication	0	0	1^^^ (3.70)	0	1 (0.52)	3 (2.06) ^§, £, &^
Postop complication	1 * (1.17)	1 ° (1.58)	1^ç^ (3.70)	0	3 (1.58)	1 (0.68) ^$^

^ 20 s asystole during colpotomy, ^§^ accidental mesenteric vessel injury, ^£^ 2 conversion into laparotomy, ^&^ bladder infraction, * Transient ischemic attack, ° Major depressive syndrome, ^ç^ periumbilical infection solved with antibiotics, ^$^ vaginal cuff bleeding.

**Table 3:** Pathologic findings at RRS.

	Cases					Controls	*p* Value
	*BRCA1*	*BRCA2*	Other Genes	Familial Risk	Total		
p53 signature *n*, (%)	7/85 (8.23)	5/63 (7.9)	5/27 (18.5)	2/15 (13.3)	19/190 (10.16)	14/145 (9.65)	NS
STIL *n*, (%)	6/85 (7.05)	6/63 (9.5)	0/27	3/15 (20)	15/190 (8.02)	18/145 (12.41)	NS
STIC *n*, (%)	5/85 (5.88)	3/63 (4.76)	0/27	0/15 (0)	8/190 * (4.21)	0/145	0.0111
Invasive carcinoma (tubal and ovarian) *n*, (%)	5/85 (5.88)	2/63 (3.1)	0/27	0/15	7/190 * (3.68)	0/145	0.0206
Neoplastic lesions (in situ and invasive) *n*, (%)	7/85 (8.23)	4/63	0/27	0/15	12/190 (6.31)	0/145	0.0015
Endometrium (EIN and cancer) *n*, (%)	2/85 (2.35)	2/63 (3.1)	0/27	0/15	4/190 (2.10)	4/145 (2.75)	NS

* Three of the patients had a concomitant diagnosis of STIC and HGSC.

**Table 4:** Clinical and histological characteristics of patients diagnosed with STIC or occult serous carcinoma.

Patient	Age at Surgery	Genetics	Mutation Type	Previous Breast Cancer	Site of Origin	STIC Present	Histology	Pelvic Washing	Ca125 at Surgery (mU/mL)	Date of Surgery	Stage	DFS (Months)	OS (Months)	Vital Status
1	49	*BRCA1*	c.5030_5033delCTAA p.(Thr1677llefs*2)	Yes	Ovary	NO	HGSC	Positive	24	31 July 2020	IIIA1	27	27	NED
2	38	*BRCA1*	c.113A>T p.(Lys505*)	No	Fallopian tubes	NO	HGSC	Negative	149	24 January 2022	IIIA1	10	10	NED
3	67	*BRCA2*	c.3939 C>G p.(Tyr1313Ter)	Yes	Ovary	NO	HGSC	Positive	74	14 March 2016	IIIC	19	79	Alive with disease
4	79	*BRCA1*	c.2075_2076 dupAT p.(Asp693Metfs*9)	No	Ovary	NO	HGSC	Positive	20	7 May 2018	IC	53	53	NED
5	56	*BRCA2*	c.3860dupA	No	Fallopian tubes	YES	HGSC	Negative	9	21 January 2019	IIA	45	45	NED
6	64	*BRCA1*	c.190T>C p.(Cys64Arg)	Yes	Fallopian tubes	YES	HGSC	Negative	22	18 January 2018	II	29	57	Alive with disease
7	58	*BRCA1*	c.5237A>C p.(His1746pro)	No	Fallopian tubes	YES	HGSC	Negative	2.9	17 October 2022	II	/	/	NED
8	49	*BRCA1*	c.2075_2076 dupAT p.(Asp693Metfs*9)	No	Fallopian tubes	N/A	STIC	Positive	9.9	30 October 2017		60	60	NED
9	59	*BRCA2*	c.del ex14-18	Yes	Fallopian tubes	N/A	STIC	Negative	4.9	18 September 2014		160	160	NED
10	66	*BRCA2*	c.700delT	Yes	Fallopian tubes	N/A	STIC	Negative	9.3	2 May 2019		41	41	NED
11	49	*BRCA1*	c.65T>C p.(Leu22Ser)	Yes	Fallopian tubes	N/A	STIC	Negative	8	1 October 2017		60	60	NED
12	56	*BRCA1*	c.5027_5045del p.(Ser1676fs)	No	Fallopian tubes	N/A	STIC	Negative	8.9	30 November 2018		47	47	NED

NED, no evidence of disease.

## Data Availability

The original contributions presented in this study are included in the article. Further inquiries can be directed to the corresponding author.
